# Periodic Catatonia Marked by Hypercortisolemia and Exacerbated by the Menses: A Case Report and Literature Review

**DOI:** 10.1155/2018/4264763

**Published:** 2018-07-04

**Authors:** Samantha Zwiebel, Alejandro G. Villasante-Tejanos, Jose de Leon

**Affiliations:** ^1^College of Medicine, University of Kentucky, Lexington, KY 40506, USA; ^2^Department of Statistics, University of Kentucky, Lexington, KY 40536, USA; ^3^University of Kentucky Mental Health Research Center, Eastern State Hospital, Lexington, KY 40511, USA; ^4^Psychiatry and Neurosciences Research Group (CTS-549), Institute of Neurosciences, University of Granada, 18971 Granada, Spain; ^5^Biomedical Research Centre in Mental Health Net (CIBERSAM), Santiago Apóstol Hospital, University of the Basque Country, 01004 Vitoria, Spain

## Abstract

Kahlbaum first described catatonia; later Kraepelin, Gjessing, and Leonhard each defined periodic catatonia differently. A 48-year-old female with catatonia, whose grandmother probably died from it, was prospectively followed for >4 years in a US psychiatric state hospital. Through 4 catatonic episodes (one lasting 17 months) there were menstrual exacerbations of catatonia and increases in 4 biological variables: (1) creatine kinase (CK) up to 4,920 U/L, (2) lactate dehydrogenase (LDH) up to 424 U/L, (3) late afternoon cortisol levels up to 28.0 mcg/dL, and (4) white blood cell (WBC) counts up to 24,200/mm^3^ with neutrophilia without infections. Records from 17 prior admissions documented elevations of WBC and LDH and included an abnormal dexamethasone suppression test (DST) which normalized with electroconvulsive therapy. Two later admissions showed CK and WBC elevations. We propose that these abnormalities reflect different aspects of catatonic biology: (1) the serum CK, the severity of muscle damage probably exacerbated by the menses; (2) the hypercortisolemia, the associated fear; (3) the leukocytosis with neutrophilia, the hypercortisolemia; and (4) the LDH elevations, which appear to be influenced by other biological abnormalities. Twentieth-century literature was reviewed for (1) menstrual exacerbations of catatonia, (2) biological abnormalities related to periodic catatonia, and (3) familial periodic catatonia.

## 1. Introduction

Catatonia is one of the six types of psychiatric disease described by Kahlbaum [1]. After the publication of his catatonia monography in 1874 [2], Seglas and Chaslin [3] proposed that catatonia was better categorized as a syndrome, while Kraepelin thought that catatonia should be included as part of the diagnosis of dementia praecox (later renamed schizophrenia) [4]. Under Kraepelin's influence, the DSM-3 considered catatonia to be a type of schizophrenia [5] but the progressive influence of Fink and Taylor [6] led to changes in the subsequent editions, until DSM-5 [7] classified catatonia as a syndrome that occurs (1) in association with another mental disorder (catatonia specifier), (2) due to another medical condition, or (3) as unspecified catatonia. The DSM-5's unspecified catatonia probably reflects two overlapping concepts: idiopathic recurrent catatonia from psychiatry in the English-speaking world [8] and periodic catatonia from continental European psychiatry.

### 1.1. Periodic Catatonia

Kraepelin and two other lesser known Europeans, Gjessing and Leonhard, are associated with the concept of periodic catatonia. Kraepelin [4] described catatonic stupor and catatonic excitement (including periodic catatonic excitement), but considered catatonia to be part of schizophrenia. Gjessing [9–22], a Norwegian psychiatrist, fully developed the concept of periodic catatonia and focused his research career on the biological abnormalities of patients with periodic catatonia. Before the introduction of psychotropic drugs, he estimated that periodic catatonia accounted for 2-3% of schizophrenia but that psychotropic drugs changed the periodicity of the symptoms [10]. Leonhard, a German, was the last member of the Wernicke-Kleist-Leonhard school [23] which developed an alternative to Kraepelin's classification of psychoses. Leonhard [24] conceptualized periodic catatonia as a type of unsystematic schizophrenia (i.e., genetically based schizophrenia) in contrast to systematic (nonperiodic and nonfamilial) types of schizophrenia. Later, German researchers following Leonhard proposed that periodic catatonia is a familial disorder and suggested some possible gene candidates; he considered it different from typical presentations of schizophrenia [25–27].

### 1.2. Biological Abnormalities in Periodic Catatonia

Rolv Gjessing [9] made many biological observations of patients with periodic catatonia, but he focused on abnormalities in nitrogen metabolism. Summarizing his life's work, his son, Leiv Gjessing, explained that these biological abnormalities did not occur simultaneously with the stuporous or excited phases; rather, they were abnormalities that could be corrected with thyroid supplementation [10]. According to Gjessing, the following biological abnormalities can be seen [9, 10]: (1) glucose abnormalities (excitement phase characterized by elevated fasting blood glucose and stuporous phase marked by elevated blood glucose in the glucose tolerance test) and (2) leukocytosis with neutrophil granulocytosis (“moderate” leukocytosis during the excited phase and “marked” leukocytosis during the stuporous phase). More recently, Lee et al. [28, 29] described mild leukocytosis in 3 of 20 patients with catatonia not complicated by medical issues.

After reviewing 13 articles [9–21] and a book [22] in English written by R. and L. Gjessing (father and son) regarding R. Gjessing's work, it is clear that their collection of longitudinal biological data is impressive although the clinical descriptions and conclusions are often opaque. The clinical description of familial periodic catatonia by Leonard and his disciples [24] is more cohesive and understandable in a contemporary context.

Table 1 describes adrenal abnormalities in 8 published patients with periodic catatonia [30–36]. Of the 8 cases (5 males and 3 females): (1) three showed no relationship between urinary steroids and catatonia [30, 32, 33], (2) one showed a decrease in urinary steroids during the excitement phase [31], (3) one showed a decrease in urinary steroids during the stuporous phase [33], (4) two cases showed an increase in urinary steroids during the stuporous phase [31, 33], and (5) one case described by McCall [34] responded to a dexamethasone suppression test (DST) with nonsuppression during stupor which normalized with recovery.

### 1.3. Catatonia and the Menses

Kraepelin [4] was the first to notice that periodic attacks of excitement in dementia praecox were sometimes related to the menses in women. Brockington, an expert in the effects of menses and psychosis [37], has published an encyclopedic review [38] of menstrual psychosis including 80 patients who were previously published by or reported to him. He highlighted 3 patients with catatonic symptoms; none of them had stuporous episodes [36, 39, 40], but 2 of them appeared to have menstrual exacerbations of epilepsy [39, 40]. Kobayashi and Kato [41] described an 18-year-old woman diagnosed with catatonic schizophrenia who had repeated catatonic stuporous episodes of premenstrual onset (7 days before). As far as we can tell, the literature describes no cases of patients with periodic catatonia in whom the onset of menses was associated with the onset of catatonia. Leonhard [24] described menstrual exacerbations in some patients with catatonic symptoms diagnosed with cycloid psychoses, but did not describe menstrual exacerbation in periodic catatonia.

### 1.4. Catatonia and Low Serum Iron

In 1991, Rosebush and Mazurek [42] first suggested that low serum iron can be a potential biological marker and possibly a contributing factor for the development of neuroleptic malignant syndrome (NMS). Later, in 1998, Lee [43] proposed that low serum iron in malignant catatonia may be a risk factor for NMS. Some authors have found that relatively low serum iron within the normal range may be associated with acute catatonia [44, 45]. Lee [28, 43] proposed that low serum iron in excited catatonia may be associated with an unfavorable benzodiazepine response.

## 2. Case Presentation

The supplemental material presents in Section S1 details concerning the assessments; 3 subsections describe: S1.1 rating scales [46–49], S1.2 laboratory studies [50–53], and S1.3 menstruation.  Section S.2 is on statistics [54–59]. The rating scales include the Bush-Francis Catatonia Rating Scale (BFCRS) and the Abnormal Involuntary Movement Scale (AIMS). Supplementary Table S1 provides the limited laboratory studies from prior private psychiatric hospital admissions. Supplementary Table S2 describes the routine laboratory studies conducted at the state psychiatric hospital including creatine kinase (CK), lactate dehydrogenase (LDH), afternoon (3 PM) cortisol, white blood cell (WBC) count, and absolute neutrophil count (ANC).  Table 2 provides a very brief summary of Supplementary Table S2. Supplementary Table S3 summarizes the descriptive analyses of the biological variables measured during the second catatonic episode.

### 2.1. Prior History

#### 2.1.1. Description of the Patient

On her first admission to our state psychiatric hospital, the patient was a 48-year-old single white female. The patient signed a consent form for a pharmacogenetic research study approved by two institutional review boards in which she provided permission for publication of her data and clinical information. Beginning at age 34, she had had 17 admissions to private psychiatric hospitals (Supplementary Table S1) during the 1980s and 1990s. During the late 1990s and early 2000s, she was admitted to the state psychiatric hospital over the course of more than 4 years (1567 days) (Supplementary Table S2). The patient was born prematurely and her mother experienced a difficult labor. She also had Rh factor incompatibility and required a blood transfusion after significant hemolysis. The outcome of these delivery complications was left-sided cerebral palsy. Although her mother died three days after childbirth, the patient experienced a stable childhood, completed college, and went on to become a teacher. At times when she did experience difficulties in her job, her aunt, the school supervisor, was available to help. She never used tobacco products, alcohol, or drugs. She had undergone several magnetic resonance imaging (MRI) studies prior to presentation, which failed to show any acute abnormalities and were remarkable only for nonspecific periventricular white matter changes.

#### 2.1.2. Prior Psychiatric Admissions

The patient's first psychiatric hospitalization occurred at age 34 for psychosis. According to the discharge summary from this first admission, the patient had been treated intermittently with benzodiazepines for two years by her family doctor. The discharge summaries from most of these 17 prior hospitalizations were obtained and summarized in Supplementary Table S1.

#### 2.1.3. Family History

The patient's paternal grandmother had died at age 37 at a psychiatric hospital, and the death certificate listed cause of death as exhaustion from toxic psychosis. She had been hospitalized for seven days prior to death. According to some of the most experienced academic psychiatrists familiar with diagnosis in the early 20th century in Kentucky, it is likely that this death certificate meant that the grandmother died of severe agitation during a catatonic episode before psychiatric medications or ECT were available. The patient's father never had any catatonic symptoms, according to family members, nor did her mother who died young three days after childbirth. Her only siblings were half-siblings.

### 2.2. Admission to the State Psychiatric Hospital

The periods between the catatonic episodes have been moved to Supplementary Section S3 and the outcome after the admission to the state psychiatric hospital to Supplementary Section S4.

#### 2.2.1. Before First Catatonic Episode Was Diagnosed by the Senior Author

The patient's mental health began to decline two months prior to admission; she became increasingly delusional and struggled to care for herself. The patient was brought to the hospital by family members after an episode of yelling and pounding on the walls and striking out at nurses at the patient's personal care home, to which she had been discharged from a private psychiatric hospital that same day. The family attempted to return the patient to that hospital but they were referred to the state hospital for long-term care. Her behavior was difficult to control, she attempted to climb out of a moving vehicle, and she was very delusional and religiously preoccupied. She was prescribed the following medications prior to the presentation to the state psychiatric hospital: diazepam up to 20 mg/day as needed, clomipramine 50 mg/day, haloperidol 20 mg/day, benztropine 4 mg/day, and divalproex sodium 750 mg/day.

The day of admission to the state psychiatric hospital in the late 1990s will be considered day 1. On that day she was described as severely agitated, screaming, combative, and aggressive, which is compatible with an excited catatonic state, but the admitting psychiatrists did not use the word catatonia. For the first several days of admission she was admitted to the acute care unit and treated with the same medications prescribed by the most recent private psychiatric hospital. During this time, her agitation was often treated with 2-mg doses of oral or intramuscular (IM) lorazepam. She was then transferred to an intermediate care unit from day 6 to day 136. All psychiatric medications except lorazepam were initially discontinued. The patient began to make improvements and olanzapine 20 mg/day was added. She had a sudden worsening of agitation on day 120 for no apparent reason, but the chart had no information on the menses. This episode of agitation was treated primarily with lorazepam as needed. On day 132, the treating psychiatrist reported that the episodes of agitation responded well to lorazepam and so he decided to add clonazepam 1 mg/day to the treatment.

#### 2.2.2. First Catatonic Episode Diagnosed and Assessed with the Bush-Francis Scale (10 Days)

On day 137, the patient was transferred to the long-term unit managed by the senior author. She exhibited no clear catatonic signs and was being treated with 1 mg/day of clonazepam and 20 mg/day of olanzapine.

On day 194, the patient started her menses. She appeared to have “overwhelming anxiety” and reported “anxiety in her body”. She was treated with 2 mg of oral lorazepam. She also had problems with eye contact, answering questions, and non-goal-directed hyperactivity, but the senior author did not realize these were initial catatonic symptoms. On day 196, the senior author realized that the patient definitively had catatonic signs (Supplementary Table S2). This first catatonic episode lasted ten days and was treated with lorazepam up to 6 mg/day added to her prior treatment of 1 mg/day of clonazepam and 20 mg/day of olanzapine.

#### 2.2.3. Second Catatonic Episode (17 Months)

During her convalescent leave, on day 338, the patient stopped taking lorazepam and began her menses the next day when catatonia reemerged. She entered a prolonged period of 17 months during which time she was treated with lorazepam and eventually ECT after much hesitation from the patient. Menses frequently exacerbated her symptoms. Supplementary Table S2 describes her symptoms and laboratory values. Testing for porphyria and syphilis was negative.

#### 2.2.4. Third Catatonic Episode (14 Days)

On day 1271 she started her menses and the next day she went to the academic general hospital for ECT but did not receive it due to a misunderstanding. On day 1276, she again presented for ECT (5 weeks after the prior ECT) but after the treatment she became extremely agitated and had to be moved to the emergency room (ER) of the academic hospital, where the senior author was called in to manage her. She was treated with lorazepam 8 mg IV and then sent to the state psychiatric hospital. Over the next 10 days she received 3 ECT treatments plus the 6 mg/day of oral lorazepam. On day 1286, the patient returned to baseline and was placed on convalescent leave.

#### 2.2.5. Fourth Catatonic Episode (4 Days)

The patient experienced one final brief episode of catatonia. On day 1563, the psychiatrist who provided ECT reported that the patient had been vomiting for 3 days and was unable to take her lorazepam, resulting in combative behavior that was managed by the family. There were no labs drawn because the patient was on convalescent leave and the catatonic episode resolved after the ECT was moved forward to day 1564 and another ECT on day 1567 was added. After those two ECTs she returned to baseline. After this last catatonic episode, the ECT was scheduled every 3 weeks and then moved to every 2.5 weeks to avoid paranoid ideation between ECT sessions. On day 1599, the patient was discharged on lorazepam 6 mg/day and ECT every 2.5 weeks and never readmitted.

## 3. Biological Abnormalities

The data on the less important biological abnormalities has been moved to Supplementary Section S5 which includes two subsections on platelets (S.5.1.) and iron supplementation (S.5.2.).

### 3.1. Exacerbations of Catatonia with Onset of Menses

#### 3.1.1. Clinical Data from the State Psychiatric Hospital

Once the relationship between the menses and the first catatonic episode was established, the family indicated, after questioning, that the patient had suffered multiple previous catatonic episodes associated with the onset of her menses. As a matter of fact, within 30 days before admission to the state psychiatric hospital (Supplementary Table S1), the patient was taken to a private psychiatric hospital for her 17th admission after the family found her at home standing in the shower for several hours, completely mute and immobile. According to the family that catatonic episode started on day 1 of the menses.

The first episode of the patient's catatonia that was observed by the senior author occurred when the patient was 49 years old and starting the first day of her menstrual cycle. It appears that the treatment at that time (20 mg/day of olanzapine and 1 mg/day of clonazepam) was not enough to prevent a catatonic relapse. Catatonia resolved by day 11 of this menstrual cycle after treatment with lorazepam. She was placed on convalescent leave and had no relapses for three menstrual cycles.

The second catatonic episode, in which catatonia reemerged and become refractory to high doses of benzodiazepines, started after the patient stopped taking lorazepam and began her menses the next day. The episode persisted for 17 months, including three regular menstrual cycles and times of irregular menses. Her menstrual cycles were sometimes three weeks apart, and other times more than sixty days apart. At other times the patient had light flow or menses for only two days at a time. Due to the association of the onset of catatonia with the menses, oral medroxyprogesterone was started in an attempt to regulate the female sexual hormone fluctuations associated with the menses. This medication was increased to 12 mg/day over the course of three months in the context of low serum progesterone levels. The patient did not show any improvement and medroxyprogesterone was stopped. The catatonic episode resolved when the patient finally agreed to proceed with ECT treatment, which appeared to help recover her response to benzodiazepines [60].

During the 12 months of convalescent leave between the second and third catatonic episodes, the menses were not associated with catatonic symptoms while the patient was on maintenance treatment with both lorazepam and ECT. The patient had a sudden lorazepam withdrawal episode due to lack of a prescription which manifested as a withdrawal seizure rather than catatonia, but this event did not coincide with the menses.

The third catatonic episode resulted from the combination of menses onset and a missed ECT treatment the next day due to a hospital misunderstanding. She recovered in 14 days after very aggressive treatment.

During the nine months of convalescent leave between the third and fourth catatonic episodes, the menses did not exacerbate any catatonic symptoms while she was on maintenance treatment with lorazepam and ECT. The last menstrual period started on day 1479 and after that she did not have any more menstrual periods. After 2.5 months of being menopausal she had a short final catatonic episode after 3 days of vomiting and probably not absorbing the oral lorazepam, which resolved with ECT.

In summary, the menses led to (1) the first catatonic episode when the patient was taking a low dose of benzodiazepines, (2) the second catatonic episode only after the patient also stopped taking lorazepam, and (3) the third catatonic episode only after the patient also was delayed in receiving ECT.

#### 3.1.2. Statistical Association between Menses and Biological Variables during the Second Catatonic Episode

The visual inspection of means and percentage of abnormal values (lower panel of Supplementary Table S3) indicated that WBC, granulocytes, and LDH did not appear to be associated with the menses. On the other hand, CK elevations appeared to have the potential to be associated with the menses since there were 7 days with menses which had a median CK of 477 U/L and 39 days without menses which had a median of 71 U/L (Supplementary Table S3, footnote 6).

Using the SPSS autocorrelation module, we verified that the assumption of independent observations was not violated and statistical tests can be used to explore significant differences in this variable (Supplementary Table S3, footnote 6). Therefore, the independent sample Mann–Whitney U test provided a p=0.002, indicating that menses was significantly associated with CK elevations.

### 3.2. CK and LDH Elevations

#### 3.2.1. Data from the State Psychiatric Hospital

During the ten days of the first catatonic episode at the state psychiatric hospital, CK values ranged from 1957 U/L on the third day of that episode (day 196) to 159 U/L on the tenth day of the episode (day 203). On the tenth day, the CK value was not yet normal despite the fact that the catatonic symptoms were no longer present. Due to the abnormal laboratory findings during this time, particularly the CK elevation of 1957 U/L, the patient was sent to the academic general hospital for further testing. She was returned to the state psychiatric hospital on day 197, with the comment that myocardial infarct had been ruled out.

The second catatonic episode lasted 17 months and was characterized by an irregular oscillating pattern of CK values. The highest value was 4920 U/L on day 372, 32 days after the onset of this second episode. From day 483 to day 721, the patient had catatonic symptoms but the CK value was within normal limits. There were no CK values during the first days of the third catatonic episode but the CK value was normal on the eleventh day of this episode (day 1282). There were no CK values taken during the fourth catatonic episode because she was not admitted to the state psychiatric hospital.

There were no LDH elevations during the first and third catatonic episodes, which were relatively short-lived. The first catatonic episode lasted only 10 days and LDH was always normal despite the peak CK elevation of almost 2000 U/L. The third catatonic episode lasted only 14 days; both the two LDH values and the CK value were normal. No CK or LDH values were studied during the fourth catatonic episode. During the 17 months of the second episode, two occurrences of LDH elevation were detected. The first occurrence of LDH elevation lasted 20 days and started on day 371, 30 days after the first CK elevated value, and at the same time CK peaked (>1,000 U/L on day 371 and almost 5,000 U/L on day 372). The second occurrence lasted only during day 411, which was the same day that CK again was > 1,000 U/L.

#### 3.2.2. Statistical Association between LDH and Other Biological Variables during the Second Catatonic Episode

A multiple linear regression model with LDH as the dependent variable and 3 independent variables, CK, WBC, and cortisol, showed good adjustment (adjusted R square =.687). The 3 variables were significant after adjusting by the others: CK (p<0.001), WBC (p=0.002), and cortisol (p=0.027). The standardized residuals showed no significant autocorrelation and partial correlations did not reach the upper and lower limits of the confidence intervals.

#### 3.2.3. Data from the Prior Psychiatric Admissions

After the arrival of the documents from most of the 17 admissions to private psychiatric hospitals, it was clear that serum CK was never measured; however, LDH elevations were described in the seventh and tenth of these 17 admissions (Supplementary Table S1). The only explanation for the elevated LDH occurred when the level reached 733 (second private hospital, tenth admission), when the physicians attributed this abnormal laboratory finding to “bruising”. The possibility of catatonia was not considered although the patient was described as very agitated and she was diagnosed with mania. In our experience at the state psychiatric hospital, the patient only developed bruises when she was severely agitated during catatonic episodes (self-induced bruises were definitively noted during the second and third episodes). Therefore, we suspect that the LDH elevation of the tenth admission in the private psychiatric hospitals was due to a severe catatonic episode with extreme agitation, comparable to the LDH elevations seen at the state psychiatric hospital during the second catatonic episode.

#### 3.2.4. Data from the Later Psychiatric Admissions

On day 3749 the patient was admitted to the community hospital with catatonic signs. A CK elevation reaching 1800 U/L led to consideration of NMS and a transfer to the university hospital. The intervention of the psychiatry department led to recovery and normalization of CK through the use of ECT and lorazepam (Supplementary Table S1).

### 3.3. Hypercortisolemia

#### 3.3.1. Data from the State Psychiatric Hospital

During the first episode of catatonia, cortisol was not directly measured. Drawn labs included hemogram, and metabolic and cholesterol panels which showed an elevated WBC of 12.3 x 10^3^/mm^3^, elevated glucose of 131 mcg/dL, and elevated cholesterol of 227 mcg/dL. After the first catatonic episode resolved, the senior author suspected that the presence of hyperglycemia and leukocytosis without infection indicated an episode of hypercortisolemia. Therefore, when the second episode of catatonia started, cortisol was measured on the first day of the episode, day 341, providing an extremely high level of 28 mcg/dL (Supplementary Table S2).

During the second catatonic episode, labs were drawn more often than during the first episode due to the confirmed suspicion for hypercortisolemia. Elevations in cortisol were found on nine separate days that labs were checked. During this second catatonic episode, 48% (23/48) of ANC and 64% (33/52) of WBC were above the normal limits. There were two periods in which the ANC increased to >16 x 10^3^/mm ^3^ and the WBC increased to >20 x 10^3^/mm.^3^ Both occurred when the cortisol values were also extremely high: >20 mcg/dL.

During the third episode of catatonia, WBC and cortisol were not measured on the first day of the catatonic episode, but the patient had a dramatic WBC elevation of 24.2 x 10^3^/mm^3^ on day 1276 which was the third day of catatonic symptoms (Supplementary Table S2); cortisol was not checked on that day. Cortisol was later measured only once, on day 1282, and was within the normal range. No labs were checked during the fourth episode of catatonia.

#### 3.3.2. Data from the Prior Psychiatric Admissions

The records from the private psychiatric hospitals indicated that DSTs had been completed twice (Supplementary Table S1). At the time (late 1980s), many psychiatric hospitals used DST as part of the workup for depression [61].

During the patient's first psychiatric admission, the DST test was performed before and after her ECT treatments. Prior to ECT, her DST revealed nonsuppression of cortisol with values of 8.7 and 9.8 mcg/dL. After ECT, her DST revealed normal suppression of cortisol with values of 0.9 and 0.8 mcg/dL. Additionally, during this admission, her WBC was found to be 11 x 10^3^/mm^3^ and glucose was elevated to 117 mcg/dL.

During the patient's eighth admission to a private psychiatric hospital, the cortisol abnormalities were so severe that Cushing's syndrome was considered at the beginning of the admission. An abnormal cortisol of 14.7 mcg/dL indicated nonsuppression and her urine free cortisol was elevated at 117 mcg/24 h. The normal range was not described in the discharge summary but this value is definitively abnormal according to McCann et al. [62], who report normal cortisol as <51.1 mcg/24 h or de Bos [63] who reports <52.5 mcg/24 h. The patient was described as having a “moon face.” In this discharge summary, her abnormal laboratory values were noted, but no further comment was made about the presence or absence of Cushing syndrome; this lack of comment suggests to these authors that Cushing syndrome was no longer considered at the time of discharge.

#### 3.3.3. Data from the Later Psychiatric Admissions

At the university hospital a WBC was found to be 17 x 10^3^/mm^3^ and abnormalities in c-reactive protein (CRP) and erythrocyte sedimentation rate (ESR) were described (Supplementary Table S1).

## 4. Retrospective Diagnosis of Catatonia Based on DSM-5 Criteria

A DSM-5 [7] diagnosis of catatonia requires the presence of three or more of the following symptoms: stupor, catalepsy, waxy flexibility, mutism, negativism, posturing, mannerism, stereotypy, agitation, grimacing, echolalia, and echopraxia. The patient exhibited (see Supplementary Table S2) stupor, mutism, negativism, posturing, stereotypy, and agitation. The DSM-5 recognizes catatonia as a specifier for various psychiatric conditions, as a disorder due to a medical condition, and as unspecified catatonia when an underlying or associated disorder cannot be easily identified. Despite her episodes of psychosis and fear, she did not meet criteria for schizophrenia or a major mood disorder. Furthermore, the extensive medical workup failed to reveal an underlying medical explanation for her catatonia. Both authors agreed with a retrospective diagnosis of DSM-5 unspecified catatonia.

## 5. Discussion

### 5.1. Case Summary

At the time of discharge, the senior author believed that the patient had an idiopathic familial type of catatonia. Her catatonia appeared to be exacerbated by her menses. The patient's menses stopped and she became menopausal during her observation period. The elevations in CK and LDH, leukocytosis, thrombocytosis, and hypercortisolemia appeared to reflect the duration and severity of her catatonic episodes.

### 5.2. CK and LDH Elevations

#### 5.2.1. Relationship of CK to LDH

At the state psychiatric hospital, CK appeared to be definitively more sensitive than LDH for monitoring the catatonic exacerbations of our patient. LDH was only elevated in the very long second catatonic episode and only occurred when CK had reached >1000 U/L. Our observations can be compared to a study [64] of professional racing cyclists, in which CK elevations occurred after a 6-day race of 800 km while LDH elevations required 20 days of racing over 2700 km. This study on cyclists suggests that LDH elevation requires the accumulation of weeks of muscle destruction. When compared with CK elevations, LDH elevations appeared to reflect greater severity and chronicity during the catatonic episode.

There were no CK values available from the 17 psychiatric admissions prior to the state psychiatric hospital admission. CK elevations cannot be ruled out since LDH elevations were documented at least twice. CK was elevated during admissions 19 and 20 and the patient was initially considered to have NMS.

#### 5.2.2. Relationship of LDH to Biological Variables Other Than CK

Our linear regression analysis with LDH as the dependent variable suggested that cortisol elevations were significantly associated with LDH elevations, although we were unable to find any specific literature that has demonstrated this association. Still, the exercise physiology literature [65, 66] describes LDH and cortisol values as markers of physiologic reactions to physical stress although they do not discuss whether they are in any way related.

#### 5.2.3. CK as a Possible Biological Marker for Monitoring Catatonia Severity

There is general agreement that CK can be used as a marker for NMS [67] and some authors propose that it can be used to monitor NMS [68]. It seems reasonable to us that CK can be used similarly in catatonia. As a matter of fact, in 1996, Northoff et al. first described elevated CK in 32 acutely catatonic patients [50]. Since the senior author treated our patient in the late 1990s, he has used CK values as an objective sign for monitoring catatonia patients [51, 52], particularly when no clinician was available to complete repeated assessments with catatonia scales for longitudinally monitoring the patient.

### 5.3. Fear and Catatonia

On the first day (day 194) that the senior author saw the patient experiencing the first signs of catatonia, he misdiagnosed her with overwhelming anxiety of unknown cause and treated her with oral lorazepam. This is not surprising since there is consistent literature on the association between catatonia and fear, reviewed by Moskowitz [69]. In his paper “Scared Stiff”, Moskowitz conceptualizes catatonia from an evolutionary point of view as a regression to response to predators in which the stuporous phase reflects the release of tonic immobility while the excitement phase reflects the fight-flight strategy. As a matter of fact, in the first description of catatonia, Kahlbaum [2] described catatonic patients as appearing as if they were “being frozen after a great fright.” One century later, Gallup and Maser [70] concluded that catatonia was a behavioral response based on “primitive defenses against predators that now misfire under conditions of exaggerated stress.” Then, Perkins [71] explained, “It seems possible therefore to view catatonia as the ultimate response to fear in a person who is under enormous psychological and physical stress and in whom regression to a primitive form of expression has occurred. The presence of organic brain disease may be necessary to lower the threshold for the release of this form of behaviour.”

Rosebush and Mazurek [72] suspect that catatonia can be treated with lorazepam because since “the catatonic patient is ‘frozen with fear,' one would expect the anxiolytic properties of lorazepam might be central to its ability to treat catatonia.” Recently Fink and Shorter [73] have proposed that “persistent fear occupies the mind of catatonic patients.”

The hypothesis that catatonia is a fear response is especially applicable to our patient because there is definitive agreement in the literature that psychological stressors induce cortisol release, particularly when a test subject feels a situation is uncontrollable or that they are being judged and evaluated by others [74]. The fear associated with catatonia could explain why our patient had hypercortisolemia during the catatonic exacerbations.

### 5.4. WBC and ANC Elevations

Although there is debate, glucocorticoid-induced elevation of ANC is thought to occur through multiple mechanisms including enhanced mobilization of neutrophils from the bone marrow, demargination of neutrophils into active circulation, and prolonged intravascular neutrophil half-life [75]. A recent study has demonstrated that glucocorticoids may alter the mechanical properties of neutrophils such that they become more mobile and are able to demarginate and circulate more easily [76]. Increased neutrophil mobility promoted by hypercortisolemia was probably an important issue in this patient since we identify dramatic changes in WBC count within hours on the same day. On day 1276 the first WBC checked in the emergency department was 24.2 x 10^3^/mm^3^ with an ANC of 19.7 x 10^3^/mm^3^. A few hours later at the state psychiatric hospital the WBC had decreased to 16.2 x 10^3^/mm^3^ with an ANC of 12.0 x 10^3^/mm^3^.

As (1) the literature definitively supports the concept that hypercortisolemia can increase WBC and (2) in this patient, during periods of high cortisol, mean and abnormal WBCs appeared higher (middle panel in Supplementary Table S3), it is clear that the WBC elevations were a sign of hypercortisolemia during the catatonic exacerbations. As there is evidence for extreme variations of WBC within a few hours (for example, day 1276), we suspect that increased neutrophil demargination was a major contributor to WBC peaks >20 x 10^3^/mm^3^.

### 5.5. Platelets

The standard laboratory upper limit for platelet count is 444,000/mm^3^, a value that this patient almost always exceeded. As the patient refused further studies, including a bone marrow biopsy, it cannot be ruled out that the thrombocytosis may have other causes independent of catatonia or that catatonia only exacerbated the underlying mechanism causing the thrombocytosis. The limited data that were available from the prior and later admissions indicated values usually within normal laboratory range or mild elevations.

The relationship of platelet count in this patient with catatonic exacerbations and hypercortisolemia remains somewhat uncertain. We were unable to find any literature supporting platelet elevations during hypercortisolemia and failed to find any significant correlations between platelet count and any other lab values. However, this patient's platelets tended to trend up with other biological abnormalities during the worst exacerbations and, more importantly, the extremely high values >800,000/mm^3^ only occurred during the very prolonged and severe second catatonic episode. Therefore, we suspect that platelet elevation was yet one more of the biological abnormalities associated with catatonia in this patient, although we are not sure of the mediating mechanism. It appears that the greatest peaks in platelet counts arose a few weeks after the highest WBC peaks. Platelet counts took more than 150 days to normalize between the second and the third catatonic episodes. So, it appears that the biological process which underlies the platelet elevations in this patient may take longer to have full effect and resolve.

### 5.6. Serum Iron Levels

The iron treatment corrected the mild iron lab abnormalities within six months but did not appear to be associated with changes in catatonic symptoms; moreover, the patient recovered only after receiving ECT. The literature reviewed in the Introduction indicates that in catatonic patients serum iron values are frequently relatively low but within normal range; their contribution to the catatonic episodes is unclear. Similarly, in our patient, it is not clear that her mild iron deficiency played any important role in exacerbation and/or recovery.

### 5.7. Menses

We suggest a two-hit hypothesis of the menses and catatonia: the patient suffered from episodes of catatonia when (1) she had onset of her menses* and* (2) she suffered from a lapse in treatment with either benzodiazepines or ECT. As detailed in Section 3.1, it is known that at times she had her menses but was not catatonic; at other times she abruptly stopped her benzodiazepines and suffered seizures but not catatonia. It therefore seems that both the lack of effective treatment and the onset of the menses were required for the patient to develop an episode of catatonia.

Kraepelin [4], Brockington [38], Kobayashi and Kato [41], Cookson [32], and Leonhard [24] described catatonic patients whose catatonia (or periodic psychoses) was influenced by the menstrual cycle. Our case represents the only example of periodic catatonia specifically exacerbated by the menses. During the second catatonic episode, there was significant CK elevation (p=0.002) during the menses, with a median CK of 477 U/L on the 7 days with menses and a median CK of 71 U/L on the 39 days without menses. No similar menstrual exacerbations could be demonstrated with other biological abnormalities, but data was limited and only available for the second catatonic episode.

### 5.8. Role of Heredity

We suspect that heredity contributed to the patient's catatonic episodes. Her paternal grandmother probably died of severe agitation during an episode of excited catatonia. Her death occurred in her 30s, which is the same age that the patient started having catatonic episodes. The father would not be expected to demonstrate catatonia exacerbated by the menses, and the siblings were only half-siblings. The patient did not have any children. Leonhard [24] and his followers in Germany [25–27] described periodic catatonia as a familial disorder; our patient probably had a form of familial periodic catatonia.

### 5.9. Limitations

This study is limited due to the uniqueness of this case and the lack of comparison; as a matter of fact, a thorough literature review failed to reveal any other case reports or case series with comparable (1) long-term follow-up and data collection, (2) presentation of catatonia exacerbated by the menses, (3) LDH elevations in catatonia, and (4) unexplained platelet elevations in catatonia. Therefore, while this case demonstrates some unprecedented findings, we have over 1,000 days of close follow-up and over 1,500 days of inpatient monitoring. Similarly, the laboratory and clinical data available from other psychiatric hospitals are certainly limited, but we have gathered information over 25 years and 20 hospitalizations.

In the late 1990s, catatonic patients were not studied for risk of pulmonary embolism using fibrin D-dimer [77]. Currently, if we had to treat and manage a patient who was catatonic for 17 months and who had platelet elevations, we would measure fibrin D-dimers to establish the risk for pulmonary embolism, which can be lethal [78, 79].

### 5.10. Suggestions for Further Studies

We have presented years of clinical observations and drawn from hundreds of biological measurements in a patient with familial periodic catatonia. This case may have implications for those patients with (1) familial periodic catatonia, (2) fear as a salient feature of catatonia, and (3) any type of catatonia.

Although familial periodic catatonia is probably rare, catatonia in general is underdiagnosed and undertreated [79], so it is unknown how many cases of familial periodic catatonia go unnoticed by clinicians. Leonhard [24] and others [25–27] describe families with periodic catatonia but do not comment on the presence of biological abnormalities. There is great need to study biological abnormalities in patients with familial catatonia.

If fear is present in at least a significant number of patients with catatonia, hypercortisolemia may be more common than is recognized in patients with catatonia. Table 1 describes the limited number of published catatonic patients examined for hypercortisolemia. If it is correct that fear is crucial in catatonia, it would not be surprising if many catatonic patients had hypercortisolemia, though this has never been systematically studied. As with our patient, some of these catatonic patients with intense fear may have leukocytosis and neutrophilia in the absence of infections. As discussed earlier, leukocytosis was described in catatonic patients by Gjessing [9, 10] and more recently by Lee et al. [28, 29].

As Section 5.2.3 describes, we propose that clinicians treating any kind of catatonic patient may consider using CK as an objective marker of severity in catatonia and that researchers should complete studies to explore this issue.

## 6. Conclusion

After a review of the literature from the early 20th century forward, we find that (1) menstrual exacerbations of catatonia, (2) biological abnormalities of periodic catatonia, and (3) familial periodic catatonia support the uniqueness of this case. Its originality is demonstrated by the long-term follow-up, including 4 years of prospective data including 4 catatonic episodes (the second one lasting 17 months) and an exhaustive study of biological abnormalities. Besides the 4-year admission at the state hospital, we gathered data from 14 years of previous admissions and from the 7 years following; these limited laboratory data also suggested that other catatonic episodes had similar biological abnormalities. Exacerbations of catatonia with the menstrual cycle have been described before but no previous description had similar detail. Moreover, during the second catatonic episode, we observed a significant CK elevation during the menses. Prior descriptions of catatonic patients included leukocytosis by Gjessing [9, 10] and Lee et al. [28, 29] and some cases of cortisol elevation by other authors [31, 33, 34], but our case is unique due to its long-term follow-up and suggests an association between hypercortisolemia and leukocytosis with neutrophilia. We have previously used CK to follow catatonic patients [51, 52], but this is the first case in which LDH not only appears to be a marker, of the severity and persistence of the muscle destruction, but also reflects the other biological processes present in the catatonic episodes of this patient.

## Figures and Tables

**Table 1:** Adrenal abnormalities in 8 published patients with periodic catatonia^1^.

First author Year	Country	Case	Age & sex	Retrospective diagnosis^2^	Endocrine measure
Ashby [30] 1952	England		38♀	Schizophrenia	Patient improved with ECT but urinary 17-ketosteroids unchanged

Gornall [31] 1953	Canada	2	36♂	Schizophrenia	↓ urinary 17-ketosteroids during excited phase, normal in interval
		3	33♀	Schizophrenia	↑ urinary steroids during stupor phase, normal in interval

Cookson [32] 1966	Canada		22♀	Schizophrenia	No relationship between ↑urinary 17-ketosteroids and stupor

Vesterggard [33] 1969	USA	1	47♂	Schizophrenia	↓ urinary steroids during stupor
		2	40♂	Schizophrenia	↑ urinary steroids during stupor
		3	29♂	Schizophrenia	No relationship between urinary steroids and stupor

McCall [34] 1989	USA		60♂	Idiopathic recurrent catatonia^3^	Lack of DST suppression during stupor, normalized with recovery

^1^Eight patients were excluded as they did not have clearly distinct episodes of periodic catatonia, including two patients described by Gunne and Gemzell [35] and six patients (Cases 1, 2, 3, 4, 9, and 10) described by Hatotani et al. [36]. Case 1 by Gornall et al. [31] and Cases 5, 6, 7, and 8 by Hatotani et al. [36] are not included among the 16 patients with measures of adrenal function, due to lack of these measures.

^2^Retrospective DSM-5 diagnoses by agreement between two of the authors.

^3^There is no family history of catatonia but the patient's symptoms appeared similar to our patient. As a matter of fact, the patient had some “preoccupation with guilt and punishment” as did our patient.

**Table 2:** Biological variables during and between four catatonic episodes at the psychiatric state hospital (admission 18).

Episode	Trigger	Duration	Symptoms		Hypercortisolemia		Muscle Enzymes		↑ Platelets^2^ (peak)
			Excitement	Stupor	↑ Cortisol^1^ (peak)	↑ WBC^2^ (peak)	↑ CK^3^ (peak)	↑ LDH^3^ (peak)	
First	Menses	10 days (194 to 203)	Yes	Yes	-	Yes (12,300)	Yes (1957)	No	Yes (598,000)

Between first and second		147 days (204 to 340)	No	No	-	-	-	-	-

Second	Menses & benzo stop	509 days (341 to 849)	Yes	Yes	Intermittent (28)	Persistent (23,300)	Yes (4920)	Yes (424)	Yes (888,000)

Between second and third		364 days (850 to 1213)	No	No	No	Initially^4^ (13,500)	No	No	Initially^5^ (510,000)

Third	Menses & ECT delay	14 days (1272 to 1283)	Yes	No	No	Yes (24,200)	No	No	Initially^6^ (474,000)

Between third and fourth		281 days (1284 to 1562)	No	No	-	-	-	-	-

Fourth	Benzo stop	4 days (1563 to 1567)	Yes	No	-	-	-	-	-

Benzo: benzodiazepines; CK: creatine kinase; ECT: electroconvulsive therapy; LDH: lactate dehydrogenase; WBC: white blood cell count.

^1^Units mcg/dL

^2^Unit count in mm^3^

^3^Units U/L

^4^WBC values were abnormal until 151 days after the end of the second catatonic episode.

^5^Platelet count was abnormal until 187 days after the end of the second catatonic episode.

^6^Platelet count was abnormal on day 5 of the third catatonic episode but after day 7 it became normal.

## Supplementary Materials

Supplementary Materials Supplementary Table 1. Summary of psychiatric admissions before and after admission number 18 to the state hospital. Supplementary Table 2. Biological abnormalities during catatonic episodes at the state psychiatric hospital. Supplementary Table 3. Statistics of biological variables during the second catatonic episode.
