# Supplementary material for: Periodic Catatonia Marked by Hypercortisolemia and Exacerbated by the Menses: A Case Report and Literature Review
Source: Case Rep Psychiatry. 2018 Jul 4;2018:4264763. doi: 10.1155/2018/4264763 (PMC6057322; doi:10.1155/2018/4264763)
Supplement: Supplementary Materials — Supplementary Table 1. Summary of psychiatric admissions before and after admission number 18 to the state hospital. Supplementary Table 2. Biological abnormalities during catatonic episodes at the state psychiatric hospital. Supplementary Table 3. Statistics of biological variables during the second catatonic episode. [file 4264763.f1.pdf]

Periodic Catatonia Marked by Hypercortisolemia and Exacerbated by the Menses:

A Case Report and Literature Review

Samantha Zwiebel, M.D., Alejandro G Villasante-Tezanos, M.Sc., and Jose de Leon, M.D.

Supplementary Material

Supplementary Text:

S.1. Assessments

S2. Statistics

S.3. Periods between Catatonic Episodes

S.4. Outcome after the State Psychiatric Hospital Admission

S.5. Additional Biological Abnormalities

Supplementary Tables:

Supplementary Table S1. Summary of psychiatric admissions before and after admission number 18 to the state hospital.

Supplementary Table S2. Biological abnormalities during catatonic episodes at the state psychiatric hospital.

Supplementary Table S3. Descriptive statistics of biological variables during the second catatonic episode.

S.1. Assessments

S.1.1. Rating Scales

The Bush-Francis Catatonia Rating Scale (BFCRS) was developed as a catatonia rating scale which includes a 14-item screening tool and a 23-item rating tool [46, 47]. Two positive findings on the screening are required to suggest the diagnosis of catatonia, while the rating scale establishes the severity of symptoms [46]. In a review of six rating scales for catatonia, the BFCRS was the most highly recommended [48].

The Abnormal Involuntary Movement Scale (AIMS) was developed to assess dyskinetic movements by scoring orofacial movements, extremity and truncal dyskinesias [49].

S.1.2. Laboratory Studies

Supplementary Table S1 provides the limited laboratory studies from prior private psychiatric hospital admissions. Supplementary Table S2 describes the routine laboratory studies conducted at the state psychiatric hospital; the more complex studies including hormones were sent out to a contracted commercial laboratory. Table 2 provides a very brief summary of Supplementary Table S2. Supplementary Table S3 summarizes the descriptive analyses of the biological variables measured during the second catatonic episode. The patient's creatine kinase (CK) was checked when the patient was at the state psychiatric hospital but was not measured during the periods of convalescent leave in which the patient was living in her own apartment. CK elevations have been described in patients with catatonia [50]; furthermore, the senior author uses CK to longitudinally monitor the severity of catatonia in his patients [51, 52]. Lactate dehydrogenase (LDH) was also measured as a marker of muscle damage, but the literature indicates that LDH is less sensitive than CK [53].

During the first catatonic episode the patient had several abnormally high values of glucose, cholesterol, and white blood cell (WBC) count that were compatible with hypercortisolemia. With the onset of the second episode, afternoon (3 PM) cortisol was used to establish hypercortisolemia. Female

sexual hormones including progesterone, estradiol, follicle stimulating hormone (FSH), and luteinizing hormone (LH) were also collected to establish menopause, but the data is not presented.

S.1.3. Menstruation

Day 1 of the menstrual cycle is defined by the initiation of menstrual flow, or menses. Menstruation or menstrual spotting was established by nursing staff and patient report during and after the first catatonic episode in the long-term unit, and by family and the patient when she was not hospitalized.

S2. Statistics

The Statistical Package for the Social Sciences (SPSS 23) was used to analyze the data. There is a long tradition in psychology of analyzing case reports with statistical methods [54-56], which is slowly moving to medicine [57]. There is no general agreement on which is the best method for establishing significant differences, and one of the major problems is that some but not all of the data from single cases can show serial dependence due to the temporal order of sequence [57]. This serial dependence can be tested using the module of autocorrelation in SPSS, which tests autocorrelations using the significance of the Box-Ljung statistics and provides partial correlations with their upper and lower limits of their confidence intervals.

S.2.1. Descriptive Analyses

The upper panel of Supplementary Table S3 describes the means, standard deviations (SDs), medians, and percentages of abnormal values of the laboratory variables: WBC, absolute neutrophil count (ANC), platelet count, CK, LDH, and cortisol, during the second catatonic episode. There were 46 days when the values of WBC, CK, LDH, and cortisol were recorded together with status of the menses during the second catatonic episode. After dichotomizing the menses into two groups of “yes” and “no,” with “light flow” or “spotting” included in the “yes” category, the values of these variables were dichotomized into two groups of "normal" and "abnormal" using the recommended laboratory ranges.

S.2.2. Mean and Percentage of Abnormal Values after Stratification by Cortisol and Menses

The second panel of Supplementary Table S3 compares the mean and the percentage of abnormal values in the group of measures with the biological variable present (high cortisol or menses) versus the group of measures with the biological variable present (low cortisol or no menses). We started with visual inspection of the data which we have summarized with a bold font in the Supplementary Table S3 to reflect which variables have potential to be associated with the stratifying variable (comparing high versus low cortisol and menses present vs. absent).

S.2.3. Testing for Autocorrelation before Testing for Significance

Then, using the SPSS autocorrelation module, we planned to verify that the assumption of independent observations is not violated before any statistical test could be used to explore significant differences after stratification.

S.2.4. Multiple Linear Regression Model for LDH

A linear regression model with LDH as the dependent variable was planned to explore whether it was associated with CK, WBC and cortisol. We planned to use the Box-Ljung tests for autocorrelation and partial autocorrelation functions to verify the lack of evidence in the standardized residual set for serial autocorrelations or complex time series structure [58, 59].

S.3. Periods between Catatonic Episodes

S.3.1. Period between First and Second Catatonic Episodes (4.5 Months)

The patient became stable with no catatonic symptoms on the combination of 20 mg/day of olanzapine, 1 mg/day of clonazepam, and 6 mg/day of lorazepam. After two successful home visits, she was placed on convalescent leave on day 264 with the same medications. On day 297 the patient came to the hospital for follow-up. She had no catatonic symptoms but she had mild dyskinetic movements in several areas (lips, jaw, tongue and upper extremities) on AIMS. As she had gone through three menstrual cycles without any

catatonic exacerbations, olanzapine was decreased and finally stopped on day 324. On day 328, during a second hospital follow-up, the patient showed no worsening of the psychotic symptoms after stopping the olanzapine and no catatonic symptoms, but she had an exacerbation of the dyskinetic movements on the AIMS (face with no movements, lips mild, jaw moderate, tongue moderate, upper extremities moderate, lower extremities mild and trunk none).

S.3.2. Period between Second and Third Catatonic Episodes (12 months)

Once the patient experienced a resolution of her catatonic symptoms, she was placed on convalescent leave. On day 1029 she ran out of her lorazepam and had withdrawal seizures four days later, but this did not trigger any catatonia. She was kept stable on 6 mg/day of lorazepam and outpatient maintenance electroconvulsive therapy (ECT) every 4 weeks at an academic general hospital.

S.3.3. Period between Third and Fourth Catatonic Episodes (9 months)

During this time of convalescent leave, the patient had no symptoms of catatonia on maintenance treatment with 6 mg/day of lorazepam and ECT every 4 weeks. The patient continued to do well through two later cycles of menses, which then became irregular. Her last known menses occurred 4 years and 1 month after admission.

S.4. Outcome after the State Psychiatric Hospital Admission

The patient was maintained on monthly outpatient ECT at the university hospital for 4 years until age 56 with no admissions. At age 59 she was admitted to a community hospital with a catatonic relapse for 13 days. When CK elevation was observed, she was transferred to the university hospital due to the possibility of neuroleptic malignant syndrome (NMS). At the university hospital, she was initially treated with bromocriptine and then transferred to the psychiatry department where she recovered with 6 ECTs and lorazepam 3 mg/day. After 52 days she was discharged on ECT every 3 weeks, lorazepam 2 mg/day and clozapine 150 mg/day (Supplementary Table S1). Then she was decreased to monthly ECT. The last ECT was given at age 60, at which time she had had 2249 days or 7 years of follow-up after the state psychiatric admission. The patient did not come back for the next monthly ECT. A sudden cardiac death

cannot be ruled out since she had recently been seen at the outpatient cardiology department of the university hospital, but the records are no longer available.

S.5. Additional Biological Abnormalities

S.5.1. Platelet Elevations

S.5.1.1. Data from the State Psychiatric Hospital. At the state psychiatric hospital, during the first catatonic episode, the patient's platelets were elevated at the peak of catatonic symptoms. During the second catatonic episode, platelets were always elevated except for one value. The first peak in platelets to a value >700,000/mm^3 occurred on day 371, while the patient was having her menses. Platelets also peaked around day 400 to day 407, with values as high as 888,000/mm^3. These latter values occurred after her menses but labs were not drawn during the first seven days of this menstrual cycle. The third peak in platelet count occurred around days 435 to 448, with a maximum of 861,000/mm^3 measured, although platelets were checked only twice during this time. Platelet count started to decrease around day 617 and continued to decrease until the end of the second catatonic episode.

During the third catatonic episode, platelets were not measured for the first four days of the episode. Afterward, most values were within the hospital's standard range during this short episode (14 days). Platelet count was not measured during the fourth catatonic episode.

The patient refused to go to the academic general hospital to be studied for her abnormal platelet count, but she agreed that her medical data could be sent to internal medicine for evaluation. The internist recommended consideration of a bone marrow biopsy but the patient adamantly refused. The psychiatrists and the patient's family agreed that a high platelet count was a minor issue compared with the risk of untreated catatonia and did not insist on the bone marrow biopsy.

S.5.1.2. Data from the Prior Psychiatric Admissions. The documentation from the private psychiatric hospitals provided little relevant information on platelet count, merely mild elevations during the sixth and tenth admissions (Footnotes 8 and 11 of Supplementary Table S1).

S.5.1.3. Data from the Later Psychiatric Admissions. The university hospital described a mild platelet elevation during admission 20 (Footnote 18, Supplementary Table S1).

S.5.2. Iron Supplementation

Iron and total iron binding concentration (TIBC) were within normal limits, but there was a low percentage of iron saturation and of low serum ferritin; thus, iron supplementation of 975 mg ferrous sulfate was added from day 622 to day 1111. Three months after starting the iron treatment, the percentage saturation remained low at 10% while the ferritin had corrected. However, this was not associated with any clinical improvement in catatonia; that did not occur until ECT was added. Between the second and third catatonic episodes, all values from the iron studies became essentially normal and iron supplementation was discontinued on day 1111.

Supplementary Table S1. Summary of psychiatric admissions before and after admission number18 to the state hospital

Age	Adm #N[1]	Days prior[2]	Days hosp[3]	Benzo-diazepine	ECT	Catatonia	DST	Other labs	Diagnosis	Other treatments (doses in mg/day)
FIRST PRIVATE PSYCHIATRIC HOSPITAL										
34	1	None[4]	15	Temazepam 30	3 times		Normalized[5]	↑WBC[6]	MD w/P	IMI 100
34	2	5	17	Temazepam ?	None				SAD, depressed	IMI 175, TXN 20
35	3	154	42		5 times				SAD, depressed	AMI 100, PER 4, TRH 4
SECOND PRIVATE PSYCHIATRIC HOSPITAL										
39	4	1377	11	Triazolam 2.5	None				MDD	AMI 100, TRF 15
39	5	32	28						SAD, depressed	HAL 40, NOR 100
39	6	63	32		None	Yes[7]		↑WBC, plat[8]	SAD	AMI?, CAR 400
39	7	87	10	Diazepam 5	None			↑ LDH[9]	SAD	AMI 150, CAR 400
39	8	6	67		None		Abnormal[10]		SAD	AMI 200, CAR 600, HAL 40
40	9	210	49	Alprazolam 1.5	None				MDD w/A, OC, P	PHE 60
40	10	42	17		None			↑ LDH, plat[11]	Mania	LiC 1800
THIRD PRIVATE PSYCHIATRIC HOSPITAL										
41	11	11	23	Lorazepam 3	None				Affective D, P	HAL 100, PER 12
42	12[11]	775	11		None				SAD w/P	AMI 100, TRF 20
43	13	255	26		None				DD, affective D	IMI 150, PER
FIRST PRIVATE PSYCHIATRIC HOSPITAL										
46	14	1147	8		None				MDD w/P	PER 16, VEN 75
46	15	2	10	Diazepam 10	None				P	FLU 20, PAR 40
48	16	429	12			Yes[12]				
48	17	40	23		None	Yes[13]		↑WBC, GLU[14]	DD, mood D	CLOI 100, HAL 20
COMMUNITY HOSPITAL										
59	19	2184	13		None	Yes		↑CK[15]	NMS	CLOZ
UNIVERSITY HOSPITAL										
59	20	0	52[16]		6 times[17]	Yes		↑several[18]	S, resolved catatonia	CLOZ 150, lorazepam 2[19]

?: dose is not known; A: anxiety; AMI: amitriptyline; CAR: carbamazepine; CLOI: clomipramine; CLOZ: clozapine; CRP: c-reactive protein; D: disorder; DD: delusional disorder; DST: dexamethasone suppression test; ECT: electroconvulsive therapy; ESR: erythrocyte sedimentation rate; FLU: fluphenazine; GLU: glucose; HAL: haloperidol; IMI: imipramine; LDH: lactate dehydrogenase; LiC: lithium carbonate; MDD: major depressive disorder; NMS: neuroleptic malignant syndrome; NOR: nortriptyline; OC: obsessive-compulsive; P: psychosis; PAR: paroxetine; PER: perphenazine; PHE: phenelzine; S: schizophrenia with; SAD: schizoaffective disorder; TRF: trifluoperazine; TRH: trihexiphenidyl; THI: thiothixine; VEN: venlafaxine; w/: with; WBC: white blood cell count.

[1]Indicates patient's N^{th} admission.

[2]Days since prior admission.

[3]Duration (in days) of that admission.

[4]She had no prior psychiatric admissions, and on admission she was not taking any psychiatric medications.
[5]Her DST results normalized after treatment with ECT. Hyperglycemia was present with glucose 117 mg/dL.
[6]WBC=11.0 x $10^3/mm^3$.
[7]Documented as: inactivity, sitting in one position for a prolonged time, unresponsive to conversation, some posturing. Catatonia was suspected.
[8]WBC=10.7 x $10^3/mm^3$ and platelets=482,000/mm^3, diagnosis of catatonia was suspected.
[9]LDH was documented as elevated, although the exact value was not available.
[10]Free cortisol level of 117 mcg/24 h was documented, and the cortisol DST was described as abnormal at 14.7 mcg/dL.
[11]LDH=733 U/L. The explanation provided for LDH in the discharge summary was bruising present at admission. Platelets = 480,000/mm^3. She was diagnosed with mania, although severe agitation during a catatonic episode is suspected by us.
[12]Discharge summary was not available. The discharge summary from hospitalization #17 states she was delusional, had catatonic behavior, and her antipsychotic medications were adjusted during hospitalization #16.
[13]Documented as: combative, withdrawn, and the family found her standing non-communicative in the shower during her menses.
[14]The patient was noted to have "an elevated white count" that "dropped to 11.1 eventually", suggesting that earlier in the admission the WBC was elevated. Additionally, she had an elevated glucose of 142 mg/dL.
[15]CK=1800 U/L
[16]The patient was first admitted to neurology for 9 days (days 3807 to 3815) and treated for neuroleptic malignant syndrome with bromocriptine. She was unresponsive to the lorazepam challenge. On day 3815 the patient was transferred to psychiatry as the CK elevation was considered secondary to catatonia.
[17]She received 6 ECTs for acute treatment. She was discharged with ECT every 3 weeks as maintenance treatment which was later decreased to every month.
[18]CK=1141 U/L, WBC=17 x $10^3/mm^3$ and platelets=465 x $10^3/mm^3$. There were also elevations of CRP 69 mg/dL (normal range 0-0.9) and ESR 45 mm/hr (normal range 0-11). Initially she also had a urinary tract infection but blood cultures were negative. Before discharge her last WBC=13.2 x $10^3/mm^3$ and ANC=9.0 x $10^3/mm^3$, indicating that they had not yet normalized.
[19]At the same time ECT was given, she received lorazepam 3 mg/day and was discharged on 2 mg/day. Clozapine was added on day 3825 and increased. She developed tachycardia and the clozapine dose was decreased from 200 to 150 mg/day at the time of discharge. She was also discharged on atenolol 2.5 mg/day, aspirin 325 mg/day, ferrous sulfate 650 mg/day and famotidine 40 mg/day.

Supplementary Table S2. Biological abnormalities during catatonic episodes at the state psychiatric hospital

Day #	Catatonic symptoms	Day of menses	Hypercortisolemia Cortisol[1]	WBC[2]	ANC[2]	Muscle enzymes CK[3]	LDH[3]	Platelets[2]
		Lab range:	(3-17)	(4.8-10.8)	(1.5-7.8)	(41-117)	(100-250)	(150-440)
	FIRST CATATONIC EPISODE (10 days)							
194	Withdrawal, mutism, staring, impulsivity, excitement	Day 1						
195	Mutism, posturing	Day 2						
196	Score of 20 in Bush-Francis scale[4]	Day 3	↑Glu[5]	**12.3**[6]	**8.5**	**1957**[7]	206	**598**
197		Day 4				**1259**		
198		Day 5		7.3		**655**		
199	Negativism, verbigeration			6.5		**333**		
200	Ambitendency, excitation							
201				6.7	4.0	**213**	212	**517**
203	No catatonic symptoms			8.2		**159**	205	**538**
	SECOND CATATONIC EPISODE (17 months)							
341	Combativeness, excitement, negativism, verbigeration	Day 1	**28**	**11.9**	**7.7**	**337**	216	**544**
342	Combativeness, excitement. stereotypy	Day 2		9.8		**532**	201	**563**
343	Score of 19 on Bush-Francis scale[8]	Day 3	16	9.1	4.3	**477**	240	**569**
347	Stereotypy, negativism, gegenhalten, withdrawal, excitement		14	9.5	5.8	**485**	214	**514**
349			16	9.2	5.9	**125**	197	
350			**12**			95		**542**
355			17	**14.6**	**10.1**	**224**	229	**612**
356	Negativism, excitement, mutism		15	**12.3**	7.5	**238**	207	**591**
358	Negativism, withdrawal		13	**13.0**	**9.1**	95	187	**635**
361	Excitement, mutism, withdrawal		14	**14.2**	**10.2**	**265**	213	**611**
362	Excitement, negativism							
363	Withdrawal, perseveration		12	**11.4**	7.4	112	227	**597**
364	Negativism, withdrawal							
365	Perseveration		**18**	**17.1**	**12.3**	78	198	**662**
368	Negativism	Day 2	12	**12.3**	**7.9**	**603**	196	**626**
369	Negativism, mutism, withdrawal, excitement	Day 3						
371	Negativism, excitement, mutism	Day 5	**22**	**23.3**	**16.8**	**1065**	**402**	**732**
372			14	**17.7**	**11.7**	**4920**	**424**	**569**
373							**255**	
375	Negativism		10	**13.2**	**8.3**	**208**	**257**	**640**
378			17			87	**243**	
384			**20**	**15.9**	**11.3**	**976**	**388**	**613**

386	Mutism, withdrawal, negativism							
389	Mutism, withdrawal		**22**	**20.0**	**16.0**	**132**	**296**	**555**
390	Excitation, withdrawal		**19**	**20.1**	**16.1**	81	**268**	**529**
391				**22.1**	**17.9**	58	**254**	**521**
393	Excitation, withdrawal	Day 1						
394	Excitation	Day 2						
396	Mutism, withdrawal	Day 4						
399	Mitgehen, mutism, excitement							
400			16	**12.6**	6.2	66	199	**888**
404			**18**	**12.7**	**8.1**	48	214	**788**
406	Excitement, stupor, staring, withdrawal and other[9]							
407	Negativism, mutism, excitement		17	**14.1**	**8.6**	39	178	**721**
410	Negativism, withdrawal, excitement, combativeness							
411	Withdrawal, autonomic abnormalities, excitement, negativism		12	**17.6**	**11.3**	**1089**	**279**	**565**
413	Improvement	Day 1	11	**12.8**	**8.6**	**266**	208	438
418			14	**14.4**		59	210	**526**
419			14	**11.6**	**8.0**	53	169	**569**
426			**18**	**15.1**	**10.9**	34	151	**663**
433			14	**13.4**	**8.0**	55	158	**655**
435			15	**15.5**	**11.2**	37	149	**861**
448			16	**13.5**	**9.6**	48	136	**766**
449	Stereotypy, withdrawal, negativism, excitement							
453	Negativism, withdrawal, ambitendency, mitgehen, mutism, bruxism							
454	Mutism, bruxism		11	**13.6**	**9.1**	**126**	144	**638**
455	Mutism, stereotypy, bruxism							
462	Mutism, other symptoms		14	5.7	3.0	**190**	220	**496**
480	Improvement in symptoms							
483			11	**11.4**	7.5	44	177	**619**
491	Withdrawal, mutism		13	**12.7**	7.0	64	156	**689**
495	Stupor, mutism, staring, stereotypy, ambitendency, withdrawal							
498	Mutism		12	**11.7**	7.4	84	152	**678**
503	Mutism		9	9.4	5.0	55	130	**593**
518	Mutism, withdrawal		6	9.7	4.7	68	133	**606**
530	Mutism	Spotting						
532	Mutism	Day 2	12	9.3	6.2	68	138	**623**
544	Mutism		**18**	9.4	4.7	75	164	**534**
561	Mutism							

603				**11.7**	**7.7**	68	139	**620**
610	Combativeness, mutism		7	9.6	5.8	77	137	**602**
617	Combativeness, mutism		7	8.3	5.0	71	132	**638**
622	Combativeness, mutism	Day 1		7.2	4.2		150	**609**
645	Combativeness, mutism		11	9.2	5.5	54	131	**580**
653	Combativeness, mutism							
666	Excitation, mutism		6	9.0	5.1	52	128	**533**
669	Excitation, mutism	Day 1						
680	Combativeness, mutism		5	8.8	4.4	69	145	**526**
701	Combativeness, mutism		6	10.5	6.5	71	194	**515**
721			4	9.0	5.0	65	148	**498**
804		Day 1						
806			**19**	8.5	6.2	55	154	**514**
849			**19**	**14.9**	**9.4**	84	177	**562**
BETWEEN SECOND AND THIRD CATATONIC EPISODES (12 months)								
888			11.3	**11.0**	**7.9**	102	164	**510**
931	No symptoms							
936			8.3	**13.5**	**9.6**	101	154	**492**
1000				9.6	5.9			**451**
1035		Spotting		7.6	4.6			**475**
1044	No symptoms							
1111				10.0			165	440
1167				7.7			144	419
1176	No symptoms							
1213	No symptoms		6.5	3.4				390
THIRD CATATONIC EPISODE (14 days)								
1272	Anxious after missing ECT	Day 2						
1274	Unexplained screaming reported by family							
1276	Serious combativeness, excitement, impulsivity			**24.2**[10]	**19.7**			**474**
1279	Excitement			9.0	5.5		140	384
1282			15.1	**11.9**	7.6	68	125	439
1283				**13.6**	**10.0**			406
FOURTH CATATONIC EPISODE (4 days)								
1563	Combative							
1567	No symptoms							

ANC: absolute neutrophil count; CK: creatine kinase; LDH: lactate dehydrogenase ; WBC: white blood count.

[1]All values are mid-afternoon levels around 3 PM. The units are mcg/dL.
[2]The units are x $10^3/mm^3$ for all blood components.
[3]The units are U/L.
[4]Gegenhalten, mitgehen, ambitendency, posturing, autonomic abnormality, perseveration.
[5]During the first episode we did not measure cortisol but the elevation of glucose to 131 mg/dL, cholesterol to 227 mg/dL, and leukocytosis suggested the presence of hypercortisolemia.
[6]Two values: 12.3 and 9.5 x $10^3/mm^3$ that day.
[7]Two other values on that day were 969 and 1575 U/L.
[8]Excitement, mutism, staring, posturing, stereotypy, mitgehen, and ambitendency.
[9]Others were stereotypy, negativism and autonomic abnormalities.
[10]Two values of 24.2 and 16.8 x $10^3/mm^3$ that day, separated by a 5-hour interval.

Supplementary Table S3. Descriptive statistics of biological variables during the second catatonic episode

S3.1.DESCRIPTIVE ANALYSES						
	WBC[1]	ANC[1]	Platelets[1]	CK[2]	LDH[2]	Cortisol[3]
Mean	12,804	8,390	610,826	294	199	13.8
SD	3,782	3,419	91,890	744	70	5.0
Median	12,450	7,803	604,000	76	183	14
Upper limit	>10,800	>7,800	>440,000	>117	>250	>17
% Abnormal	67% (31/46)	50% (23/46)	98% (45/46)	35% (16/46)	17% (8/46)	25% (11/44)
S3.2. MEAN AND % OF ABNORMAL VALUES AFTER STRATIFICATION						
Cortisol	WBC	ANC		CK	LDH	
Low (N=35)						
mean	**11,711**[4]	**7,256**[4]		*303*[5]	**187**[4]	
% abnormal	**60% (21/35)**	**41% (14/34)**		*37% (13/35)*	**9% (3/35)**	
High (N=11)						
mean	**15,354**[4]	**10,872**[4]		*270*[5]	**214**[4]	
% abnormal	**82% (9/11)**	**82% (9/11)**		*36% (4/11)*	**36% (4/11)**	
Menses						
No (N=39)						
mean	*12,579*[5]	*8,130*[5]		**264**[4,6]	*194*[5]	
% abnormal	*67% (27/39)*	*50% (19/38)*		**28% (11/39)**	*15% (6/39)*	
Yes (N=7)						
mean	*12,600*[5]	*8,180*[5]		**472**[4,6]	*231*[5]	
% abnormal	*57% (4/7)*	*43% (3/7)*		**86% (6/7)**	*14% (1/17)*	

ANC: absolute neutrophil count; CK: creatine kinase; LDH: lactate dehydrogenase; WBC: white blood count.

[1]Units of blood component counts are $/mm^3$.

[2]Units are U/L.

[3]Units are mcg/dL.

[4]**Bold font** is used to describe that both the mean and the percentage of abnormal values were consistently and clearly higher in the group of measures with the biological variable present (high cortisol or menses) versus the group of measures with the biological variable absent (low cortisol or no menses).

[5]*Italic font* is used to indicate that the mean and the percentage of abnormal values were neither consistently higher nor clearly higher in the group of measures with the biological variable present (high cortisol or menses) versus the group of measures with the biological variable absent (low cortisol or no menses).

[6]CK showed no significant autocorrelation (all Box-Ljung statistics were non-significant; p ranged from 0.163 to .990) and all partial correlations were extremely low (range: 0.92 to 0.178) and very far from the upper and lower limits of the confidence intervals. This indicates that the assumption of independent observations was not violated and statistical testing can be used to explore significant differences in this variable. A Mann-Whitney test comparing ranks of days with and without menses provided a significant result (p=0.002). The median in the 39 days with no menses was 71 and the median in the 7 days with menses was 477.
